# Barriers and facilitators of adherence to low-dose aspirin during pregnancy: A co-produced systematic review and COM-B framework synthesis of qualitative evidence

**DOI:** 10.1371/journal.pone.0302720

**Published:** 2024-05-03

**Authors:** Raya Vinogradov, Eleanor Holden, Mehali Patel, Rowan Grigg, Linda Errington, Vera Araújo-Soares, Judith Rankin

**Affiliations:** 1 Population Health Sciences Institute, Newcastle University, Newcastle, United Kingdom; 2 National Institute of Health and Care Research Applied Research Collaboration North East and North Cumbria, Newcastle, United Kingdom; 3 Research Directorate, Newcastle upon Tyne NHS Hospitals Foundation Trust, Newcastle, United Kingdom; 4 Public Contributor and Expert by Experience, London, United Kingdom; 5 Stillbirth and Neonatal Death Society (Sands), Charitable Organisation, London, United Kingdom; 6 Action on Pre-eclampsia (APEC), Charitable Organisation, Evesham, United Kingdom; 7 Medical Faculty Mannheim, Division of Prevention, Center for Preventive Medicine and Digital Health (CPD), Heidelberg University, Heidelberg, Germany; Universita Politecnica delle Marche, ITALY

## Abstract

**Introduction:**

Women at increased risk of developing pre-eclampsia are advised to take a daily low-dose of aspirin from 12 weeks of pregnancy to reduce their risks. Despite the well-established prophylactic effect of aspirin, adherence to this therapy is low. This systematic review aimed to summarise evidence on the barriers and facilitators of adherence to low-dose aspirin to inform intervention development to support decision making and persistence with aspirin use for pre-eclampsia prevention.

**Materials and methods:**

A systematic review and meta-synthesis of qualitative research was co-produced by representatives from charities, and public, clinical and academic members. Eight electronic databases (MEDLINE, PsycINFO, CINAHL, Web of Science, Scopus, EMBASE, Prospero, OpenGrey), archives of charities and professional organisations were searched (between October and November 2023 and re-run in August 2023) using predefined search terms. Studies containing qualitative components related to barriers and facilitators of adherence to low-dose aspirin during pregnancy were included. Quality assessment was performed using the Critical Appraisal Skills Programme checklist for qualitative research. A combination of the COM-B framework with phases of adherence process as defined by international taxonomy was used as the coding framework. Co-production activities were facilitated by use of ‘Zoom’ and ‘Linoit’.

**Results:**

From a total of 3377 papers identified through our searches, five published studies and one dissertation met our inclusion criteria. Studies were published from 2019 to 2022 covering research conducted in the USA, Canada, UK, Netherlands and Australia. Barriers and facilitators to adherence were mapped to six categories of the COM-B for three phases of adherence: initiation, implementation, and discontinuation. The discontinuation phase of adherence was only mentioned by one author. Four key themes were identified relating to pregnancy: ‘Insufficient knowledge’, ‘Necessity concerns balance’, ‘Access to medicine’, ‘Social influences’, and ‘Lack of Habit’.

**Conclusions:**

The COM-B framework allowed for detailed mapping of key factors shaping different phases of adherence in behavioural change terms and now provides a solid foundation for the development of a behavioural intervention. Although potential intervention elements could be suggested based on the results of this synthesis, additional co-production work is needed to define elements and plan for the delivery of the future intervention.

**Trial registration:**

PROSPERO CRD42022359718. https://www.crd.york.ac.uk/prospero/display_record.php?ID=CRD42022359718.

## Background

Pre-eclampsia (PE) is a pregnancy related syndrome that occurs in 2%-5% of pregnancies and can lead to devastating outcomes such as maternal and fetal death [1]. PE is the second leading cause of maternal mortality [2] with an estimated global death-toll of 60,000 per year [3], and one of the leading causes of stillbirth and preterm delivery [4]. Short-term healthcare costs of caring for a mother and baby affected by PE are double those of an uncomplicated pregnancy [5]. In the long-term, PE contributes significantly to cardiovascular morbidity amongst women [6,7]. Currently, the only treatment for PE is delivery of the baby, and therefore prediction and prevention of the disease remains a high priority [4,8].

Women that are deemed to be at increased risk of developing PE are offered prophylactic low-dose aspirin from 12 weeks of pregnancy [9,10]. This is based on a meta-synthesis of data from numerous trials [11–13] wherein some clinical trial cohorts have demonstrated notably high adherence levels: Mone et al. reported a 95–96% adherence rate [14], and Rolnik et al. found adherence exceeding 85% in 79.9% of participants [15]. However, when examined outside the controlled environment of clinical trials, adherence rates have generally fallen short of those observed within the trials, with non-adherence rates ranging from 46% to 94% [16,17]. Recently, the Combined Multimarker Screening and Randomized Patient Treatment with Aspirin for Evidence-Based Preeclampsia Prevention (ASPRE) trial demonstrated the importance of adherence to aspirin; women with adherence of ≥ 90% had lower odds of developing PE than women with adherence < 90% (OR 0.24 (95% CI 0.09–0.65) vs 0.59 (95% CI 0.23–1.53)) [18]. This means that women with a higher degree of adherence benefited from aspirin nearly two times more than those that did not adhere to aspirin as well. Thus, low adherence in a high-risk population is likely to substantially reduce the effectiveness of aspirin prophylaxis [18].

Further the WHO recognises the impact of non-adherence on a health care system and population health [19,20]. The report highlights that younger people (less than 25 years of age), women, people from low socioeconomic backgrounds, those with low literacy levels, and the unemployed may struggle to adhere to long-term therapy. Addressing this issue is crucial to ensure that aspirin prophylactic treatment effectively benefits those most in need of the intervention [19]. This is particularly significant given the absence of any alternative risk reduction strategy.

A clear understanding of the barriers and facilitators of adherence to aspirin prophylactic treatment is needed to help to develop behaviour change intervention to support women to make decisions about aspirin use and to adhere to advised schedule of medicine intake. This systematic review and meta-synthesis was conducted with the aim of identifying, appraising and synthesising the qualitative evidence related to barriers and facilitators of adherence to low-dose aspirin during pregnancy to enrich understanding of the determinants of prophylactic medication taking in pregnancy.

## Methods

A co-production approach to the systematic review and meta-synthesis was applied as a way of inclusive, collaborative, and creative working. The use of a co-productive approach is recognised to have the potential to achieve higher impact as key stakeholders are involved throughout the entire research journey from knowledge creation to knowledge translation [21]. This review was approached with the highest possible degree of involvement. Key stakeholders were involved in question formulation, planning methods, protocol writing, developing the search strategy, conducting searches, selecting studies, collecting data, assessing risk of bias, data analysis, interpretation of findings, writing for publication, and will be involved in other forms of dissemination of the results. This level of involvement in a systematic review has been described by the ‘Authors and Consumers Together Impacting on eVidencE’ (ACTIVE) framework of involvement of stakeholders as ‘leading’ [22].

The review team consisted of public, charity, clinical and academic members which enabled access to a wide range of expertise and experience. Public contributors were enrolled via purposive recruitment with the aim to support the overarching ‘Aspirin in pregnancy’ project that will continue beyond this systematic review work. Representatives from two key charities, Sands (the Stillbirth and Neonatal Death Society) and APEC (Action on Pre-eclampsia), as well as a member of the public with experience of PE and use of aspirin in a subsequent pregnancy, co-led this review alongside an obstetric sonographer, medical librarian, a professor of maternal and child health and a professor of prevention with expertise on complex intervention development and behavior change.

Co-production methods were applied alongside Preferred Reporting Items for Systematic Reviews procedures [23], PRISMA check list is available in S1 File. Co-production activities were facilitated by use of ‘Zoom’, a widely used video conferencing service, and free public online space ‘Linoit’ (linoit.com), an online collaborative whiteboard platform. Both platforms do not require users to download or install a specific software program to enable use, providing accessible web space across a full range of operating systems. ‘Zoom’ facilities were used by the co-production team to facilitate group meetings while ‘Linoit’ was used to facilitate synthesis of the data.

The review was registered in the International Prospective Register of Systematic Reviews systemic review database (registration number CRD42022359718).

### Training

All lay co-production team members were novice to the systematic review methods and were trained by the first author with clinical academic experience. Training included introduction to co-production and systematic review supplemented by Cochrane on-line training and A Research Handbook for Patient and Public Involvement [24], screening and ‘Rayyan’ tool training of a mock database of articles, quality appraisal and data extraction training. Additional support was provided by encouraging reflection and continuous communication with the rest of the group during all stages of the review.

### Search strategy

The review question, ‘What are the barriers and facilitators of adherence to low-dose aspirin during pregnancy’, was guided by a modified PiCO (Population of interest, Context, Outcome) framework to suit qualitative research [25,26]. For the purpose of this review, aspirin use for the prevention of pregnancy complications for any placenta mediated disease was considered. Studies containing a qualitative component inclusive of mixed methods, interviews, surveys, focus groups and ethnographies were considered for inclusion. Although study searches were conducted in English in English-based databases, no language restriction was applied during title and abstract screening. Searches were not time limited and were conducted from inception of the databases to 25.08.2023. This decision was based on our preliminary searches conducted on MEDLINE which was limited to publications from 1980 to 2022 vs publications from the inception of the database to 2022 using the same search strategy. The preliminary searches identified 11 studies published prior to 1980 and therefore the decision was made not to limit searches to a publication date. No restriction to the study setting or country was applied.

Search terms were co-produced in a group meeting capturing all possible aspects that may link to adherence in lay terms, as well as link with theories of behaviour explaining adherence. Using a predefined search strategy available in S2 File, searches were undertaken between August and August 2023 in MEDLINE, PsycINFO, CINAHL, Web of Science (WoS) and Scopus, EMBASE, Prospero and OpenGrey to include research reports, dissertations, and conference papers. Websites and archives for professional and charitable organisations such as the Royal College of Obstetrics and Gynaecology, Royal College of Midwifery, Pre-eclampsia Foundation, APEC, and SANDS were searched, and key charities were contacted [27]. A wide choice of databases was dictated by the need to capture all possible published and unpublished literature. Medline was selected as the most comprehensive source of biomedical literature. PsycINFO was chosen for its likelihood to include specific studies in behavioral science. CINAHL was selected for its likelihood to include publications related to nursing and allied health practices. Web of Science and Scopus were utilized to capture the breadth of scientific subjects. EMBASE was chosen for its likelihood to include studies related to the use of medicines. Prospero was specifically used for searching for systematic reviews. Finally, OpenGrey allowed access to grey literature.

Citation searches were performed for all included articles. Google and Google Scholar platforms were utilised to support grey literature and citation searches. Once deduplicated in the EndNote X9 software, all titles and abstracts were transferred to ‘Rayyan’, a web-based online tool, for double screening by RV, LE, EH and MP whilst authors were blinded to each other’s decisions. Screening was performed using predefined inclusion/exclusion criteria available in S3 File. We included papers with abstracts in English language targeting use of aspirin for prophylactic reasons, primary qualitative or mixed-methods studies focusing on experiences of women/pregnant people in any settings and of any age. While we excluded studies lacking a qualitative component, focusing on therapeutic use of aspirin or use or prophylactic aspirin in pre-conceptional or postnatal periods. Literature reviews such as narrative and systematic reviews as well as editorials, commentaries or educational materials were excluded from this review. There was a small proportion of disagreements when screening for inclusion mainly related to the methodology type and those were resolved in a consensus meeting. All potentially relevant titles and abstracts progressed to full text reviews. Full reviews were conducted by two authors independently and all ambiguous texts were discussed with a third author (JR). Citation tracking was performed for all included texts.

### Quality assessment and data extraction

Several appraisal tools were reviewed by the public contributors in a joint meeting (RV, LE, EH and MP) and the Critical Appraisal Skills Programme (CASP) checklist for qualitative research [28] was selected due to its ease of use. All included texts were assessed by two authors blinded to each other’s judgment and subsequently any discrepancies were resolved in a consensus meeting during which the studies were reassessed and discussed. An additional process was implemented to check uncertainties by engaging in reflective discussions with senior academic members (VAS and JR). No additional exclusions were made based on the quality assessment.

Data were extracted by two reviewers independently (RV and EH). Bibliographic, methodology and population data were extracted into a pre-designed and piloted Excel worksheet. For this analysis, we considered both first level (direct quotes of participants) and second level constructs (authors’ interpretation) to be eligible for the analysis. This approach allows for inclusion of voices of the researchers who were directly embedded in the individual projects adding to a multidimensional interpretation. Data were extracted from the result sections, tables, figures, and supplementary materials, and imported in respective files into QSR NVIVO V.12 software for data management. Linoit and Microsoft Excel were utilized when working with public contributors.

### Synthesis

Framework synthesis was selected as the method of choice for the synthesis of qualitative data for this systematic review. This methodology arose from a Framework analysis approach developed for analysis of primary studies [29] and later extended to a level of meta-synthesis of qualitative research used in health care settings [30,31]. Framework synthesis is used in the context of the development of complex interventions, offers flexibility to explore and test existing theories and also welcomes stakeholder contribution [32], therefore this approach fits well with the aim of this project.

The following five stages of the framework analysis [33] aligned to a systematic review process [32] were used:

1. Familiarisation stage aligned to the stage of research question formation and scoping of the literature.
2. Framework selection stage consisting of selection of initial framework drawn from scoping of the literature.
3. Indexing stage is aligned to screening, quality assessment and data extraction.
4. Charting is aligned to the synthesis stage of the review process and consisting of coding and grouping the primary data.
5. Mapping and interpretation stage is aligned to the final stage of the synthesis when data are interpreted to answer the review question.

The coding framework selected for this synthesis is based on the COM-B framework [34] with elements of the international taxonomy for adherence to medication [35] to ensure alignment to the phases of the adherence process as illustrated in Table 1. The COM-B consists of three interlinked domains: capability, opportunity, and motivation [36]. Each domain in the COM-B is subdivided further to two: referring to physical or psychological capability, social or physical opportunity, and automatic vs reflective motivation. The COM-B framework postulates that motivation is likely to arise in the presence of capability and opportunity and behaviour change will only occur when all three conditions are optimal (capability, motivation and opportunity [36–38]. The COM-B is used as a diagnostic tool that can identify what needs to change in order to induce desired behavioural change. Therefore, the selection of this framework is justified by an overarching aim of this project to gain in-depth and detailed understanding of barriers and facilitators related to adherence behaviour that would subsequently aid the development of a behavioural intervention.

**Table 1:** Coding framework based on a combination of the COM-B framework and phases of adherence process.

COM-B	Capability		Opportunity		Motivation	
Phases of adherence process	**Physical** *(Physical skill*, *strength or stamina)*	**Psychol.** *(Knowledge or psychological skills*, *strength or stamina to engage in the necessary mental processes)*	**Social** (*Opportunity afforded by interpersonal* *influences*, *social cues and cultural norms* *that influence the way that we think about* *things)*	**Physical** *(Opportunity afforded by the environment involving time*, *resources*, *locations*, *cues*, *physical affordance)*	**Automatic** *(Automatic processes involving emotional reactions*, *desires (wants and needs)*, *impulses*, *inhibitions*, *drive states and reflex responses)*	**Reflective** *(Reflective processes involving plans and evaluations)*
Initiation						
Implementation						
Discontinuation						

*Text in italic extracted from ‘The Behaviour Change Wheel A Guide to Designing Interventions’ book by Michie et al, 2014 [36].

The COM-B consists of three interlinked domains: capability, opportunity, and motivation [36]. Each domain in the COM-B is subdivided into further two: referring to physical or psychological capability, social or physical opportunity, and automatic vs reflective motivation. The COM-B framework postulates that motivation is likely to arise in the presence of capability and opportunity and behaviour change will only occur when all three conditions are optimal (capability, motivation and opportunity [36–38]. The COM-B is used as a diagnostic tool that can identify what needs to change in order to induce desired behavioural change. Therefore, the selection of this framework is justified by the overarching aim of this project: to gain in-depth and detailed understanding of barriers and facilitators related to adherence behaviour that can, subsequently, aid the development of a behavioural intervention.

While the whole co-production team was involved in stages one (familiarisation) and five (mapping interpretation), the framework selection stage was led by two researches familiar with the field of adherence to medicines and behavioral change (RV and VAS), and stage four (charting) was conducted by two researches (RV and EH) in a series of on-line meetings. Charting and mapping activities were facilitated by ‘Linoit’ platform that allowed users to create and share digital sticky notes in real time for categorisation and organisation of the content, example of such activity is available in S5 File.

## Results

**Fig 1:**
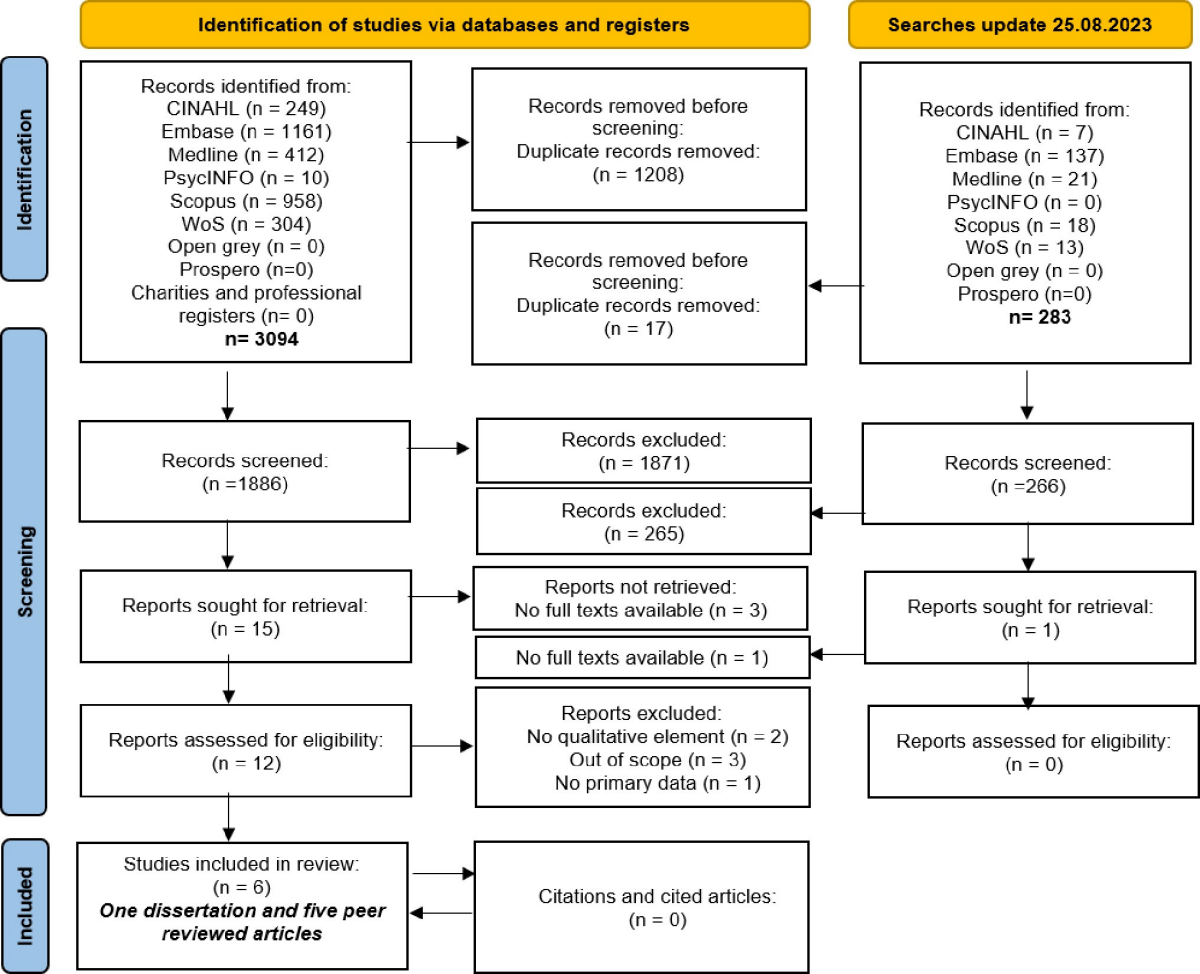
PRISMA diagram.

After screening 3,377 titles and abstracts, 12 full reports were assed for eligibility. Six reports were excluded due to lack of a primary data [39], being out of scope for this review [40–42] or having no qualitative element [43,44]. Six studies met the inclusion criteria and were eligible for inclusion in this systematic review as illustrated in Fig 1; one Master’s degree dissertation and five studies published in peer reviewed journals were included.

Included work was produced between 2019 and 2021 and studies were based in the US, Canada [45], UK [46–48], The Netherlands [49], and Australia [50]. Four studies employed purely qualitative approaches [46–49]; one used mixed methods [50] while one had a separate qualitative component [45]. Different qualitative data collection approaches were used including textual data [45], interviews [46–48,50], and focus group discussions [49]. Reflective of the methods for data collection, sample sizes varied from 6 to 807. All but one study population comprised women from an increased risk group of PE, while one study by Vestering et al recruited women from a low-risk group. Five studies investigated exclusive use of low-dose aspirin (doses were not always explicitly mentioned), while Vestering et al investigated the use of a polypill, a pill combining low-dose aspirin and calcium. Adherence levels within study populations ranged from 100% adherence to complete non-adherence. All studies were of a reasonable quality with most common deficiencies related to reflectivity aspects of the authors and rigor of the qualitative data analysis reported in the studies using both qualitative and quantitative methods. Summary of included studies are presented in Table 2 while quality assessment of the included studies can be found in S4 File.

**Table 2:** Summary of the included studies.

Author	Year of publication	Country	Data collection	Medication and dose	Population type	Number of participants	Adherence levels	Timing of the interview
**Ahmed et al [45]**	2021	US, Canada, UK, other countries	An optional text box within questionnaire	Low-dose aspirin (exact dose not specified)	High-risk	807	NK	Not stated
**Fenn et al [46]**	2019	UK	Semi-structured interviews	Aspirin (75 mg)	High-risk	13	70–100%	Gestational age of 26 and 36 weeks
**Shanmugalingam et al [50]**	2020	Australia	Interviews	Aspirin (100–150 mg)	High-risk	6	>90% and <90%	12 months post-partum
**Vestering et al [49]**	2020	Netherlands	Focus groups	Polypill (Calcium + LDA)	Low risk	25 women (7 focus groups)	NA	Gestational age of 8 to 24 weeks
**Vinogradov et al (1) [47]**	2021	UK	Semi-structured interviews	Aspirin (75 mg)	High-risk	14	0–70%	4–18 months post-partum
**Vinogradov et al (2) [48]**	2021	UK	Semi-structured interviews	Aspirin (75 mg)	High-risk	14	0–70%	4–18 months post-partum

Here we provide a detailed account of the results for the three phases of adherence structured by the COM-B constructs.

### Initiation of low dose aspirin

**Fig 2:**
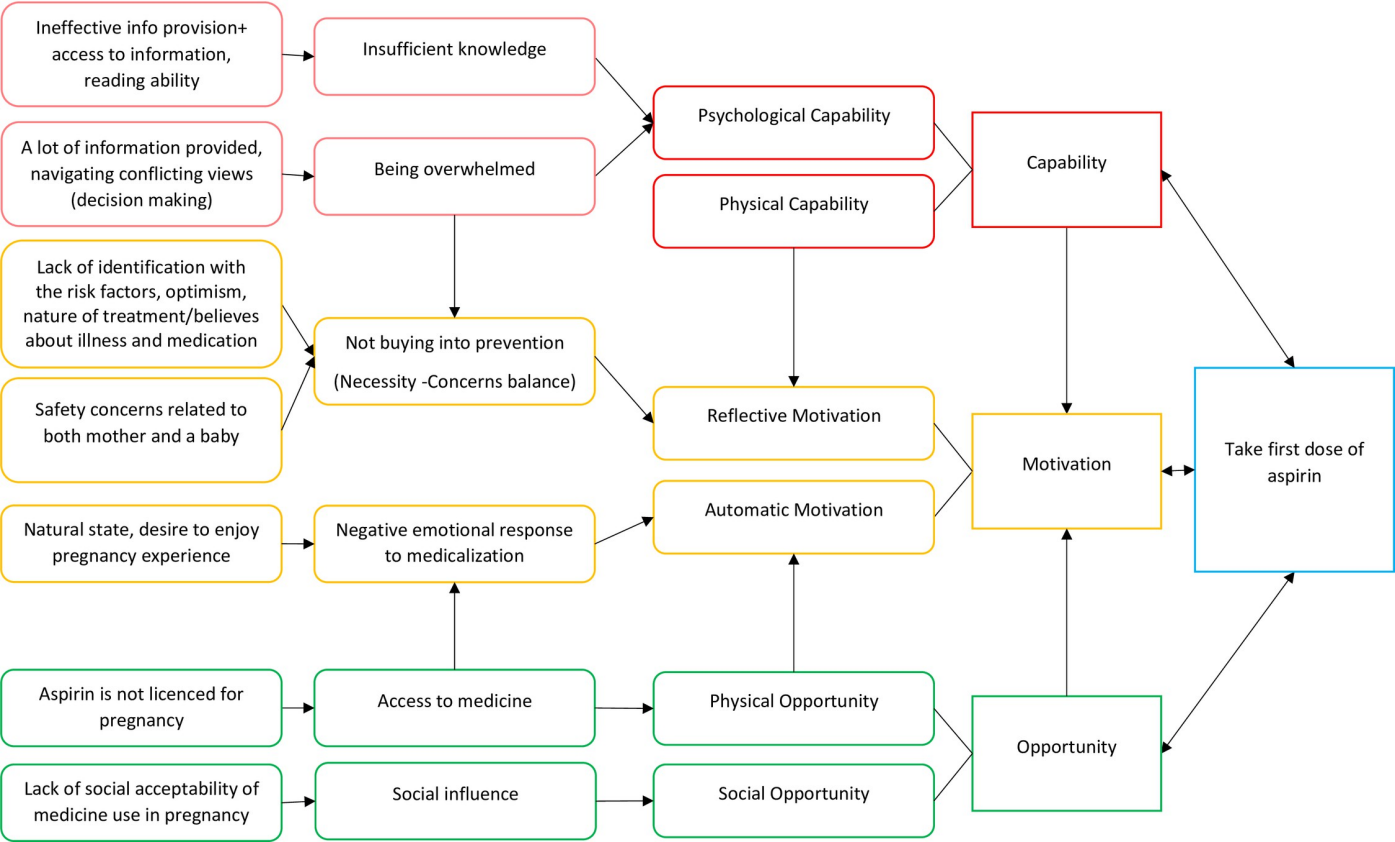
Barriers to initiation of aspirin.

The initiation phase of adherence is defined as the intake of the very first dose of aspirin for the purpose of PE prevention. The main themes related to the initiation process are illustrated in Fig 2.

### Psychological capability: Barriers

This category appears to be particularly loaded with data providing granularity and depth to our understanding of the issues women face and signaling the magnitude of the difficulties women face when considering use of aspirin during pregnancy as a prophylactic measure.

Two themes were allocated under psychological capability: ‘Insufficient knowledge’, and sense of ‘Being overwhelmed’.

### Insufficient knowledge

Data suggested that lack of knowledge about the disease and aspirin in the context of prevention of PE, prevents women from being able to decide about aspirin use [46–49]. A reduced ability to read and ineffective information provision led to reduced opportunities to gain essential information required for decision making [47]. Women reported that few resources are provided to help them to access appropriate information related to the use of aspirin in pregnancy; in some instances, information seemed to be provided to some women while others could not recall having had any [46,47,49].

*‘‘Most women had limited knowledge of pre-eclampsia. Women who knew someone with a history of pre-eclampsia considered it a serious condition for both mother and child*. *However*, *others said they had been unaware of the severity of the complications and incidence so far*.*” [49]*

*“I know it’s painkiller*, *pain relief*, *it gets rid of headache*, *it thins your blood*. *And I know if you are going to have a heart attack then stick one under your tongue*. *But when they prescribe these things*, *they don’t tell what they exactly for*.*” [47]*

*“It doesn’t strike me in my memory that I had much written information about it (aspirin) which I think for me that would be helpful*.*” [47]*

### Being overwhelmed

While decisional processes at this phase required use of information; women were overwhelmed by the amount of new information provided to them in first trimester of pregnancy generally [47–50]. The consistency of information regarding the use of aspirin varied among healthcare professionals from different professional backgrounds [48,50]. Conflicting information encountered by women necessitated a high level of cognitive ability and skills from women to navigate conflicting advice [47,50].

*‘‘Women also mentioned there was an ‘information overload’ (or a cognitive overload) during consultations with obstetricians and midwives*, *preventing women from being able to effectively consider the relevant issues and make an informed decision*.*” [48]*

*‘‘A lot of people aren’t fantastic readers either. There are still people who can’t read very well*. *To some people it’s just a lot of scribble on paper*.*” [47]*

### Psychological capability: Facilitators

In addition to the barriers, three themes were identified as facilitators within the psychological capability category: ‘Prior knowledge’, ‘Consistent messaging’, and ‘Information provision in easy-to-understand and to process format’.

### Prior knowledge

Good knowledge about aspirin use for PE prevention was reported to be beneficial with women commencing therapy even prior to seeing an obstetrician [46].

*‘‘Several of the women had extensive knowledge regarding aspirin therapy prior to their appointments*, *either due to doing their own research around aspirin*, *information received following their previous pregnancy or due to their occupation*.*” [46]*

### Consistent messaging

Consistency of messaging from numerous sources had positive impact on aspirin uptake.

*“When I was told by the first doctor*, *I was still a bit skeptical and it’s only when I saw the second and third doctor*, *it sunk in and I thought*, *it must be important as they are all saying the same thing*. *It then made sense*. *It works well when doctors communicate the same thing*, *it gives us confidence*.*” [50]*

### Information provision in easy-to-understand and to process format

Ability to access alternative formats of information was proposed as a potential facilitator by women suggesting that information provision should not be one-size fit all. Women suggested that booklets, leaflets as well as videos could be used to provide information needed to decide about use of aspirin in pregnancy. The potential benefit of the delivery of information in bite-sizes, is that it is easy to understand and process as suggested by Vestering et al and Vinogradov et al [47,49].

*(47)‘‘Women preferred information to be provided in a layered fashion as they could choose themselves how much (more) detail they wanted to know*.*” [49]*

*‘‘Women felt that information should be delivered in an easy to understand and accessible way*. *‘‘ [47]*

### Social opportunity: Barriers

#### Social influence

‘Social influence’ was a key theme and presented as a barrier under the category of social opportunity. Lack of consistency amongst health care professionals (HCPs) from different professional groups [48,50] and poor information provision left women to look for support somewhere else, finding information online using online social networks. Women often found views that were confirmatory to their pre-existing beliefs rather than extending their knowledge [47,48].

*‘‘ I did read a forum cos I’d Googled aspirin during pregnancy and there was a lot of mixed people…And someone had actually wrote the same thing that I thought why pharmacies are reluctant to give aspirin to pregnant people*.*” [48]*

#### Social opportunity: Facilitators

The Social opportunity category for initiation yielded two facilitative themes: ‘Rapport and trust with HCP’, and ‘Social comparison and support’.

#### Rapport and trust with HCP

Reports of good relationships with HCPs seemed to be an important factor correcting for confusion caused by conflicting information received from other medical or alternative sources [46,47,50].

*“I kind of trust what doctors and medical staff tell me so erm*, *the fact that I was told*, *to me*, *it would probably help*, *or not do any damage if it didn’t help*, *erm*, *that was enough for me really*.*” [46]*

#### Social comparison and support

Positive social comparison by means of social media provided a good opportunity for social support much looked-for by women [47,50].

*‘‘Women elaborated on the positive impact of social media in reassuring them on the use of aspirin in pregnancy: “Speaking to other women that have been through it (pre-eclampsia) and that are going through it–you know finding friends who are on or who have taken aspirin in pregnancy*, *who are going through similar things gave me comfort in taking it*.*” [50]*

### Physical opportunity: Barriers

#### Access to medication

A key barrier related to physical opportunity during initiation was ‘Access to medication’. Like many existing medications, aspirin is not licensed for use during pregnancy. Therefore, even though this drug may seem readily available, it can only be prescribed by a healthcare provider. However, aspirin could be advised and not prescribed, creating difficulties to access the medication and confusion causing women to re-think their decision about use of aspirin (linked to reflective motivation below).

*“The chemist kept telling me that I should not take aspirin while I was pregnant despite my doctor’s advice*.*” [50]*

*‘‘I got the prescription on the day I had my 12 weeks scan and I took it to the pharmacy and they said it wouldn’t be available until the next day and I didn’t have any way to get there and I never ended up picking it up … I think it was after my 20 week scan*.*” [48]*

### Automatic motivation: Barriers

#### Desire to enjoy the pregnancy experience

The automatic motivation category relates to automatic unconscious processes that can be described as unintentional, efficient, uncontrollable, or unconscious [51]. Examples of such responses include emotions, impulses, habits, and inhibitions. In this review, a key driver to an automatic response was a ‘Desire to enjoy the pregnancy experience’ leading to a negative response to the idea of medicalisation at the emotional level [47–49].

*‘‘Many women were explicit about their passive approach because of desire to enjoy the pregnancy …” [47]*

*‘‘If I was given a prescription*, *I must have put it straight in the bin*.*‘‘ [48]*

#### Reflective motivation: Barriers

Cognitive motivation refers to explicit and controlled processes employed by women. Those processed were defined by being intentional, required use of cognitive resources, ability to stop voluntarily, and operate within conscious awareness [51].

#### Necessity concerns balance

Barriers within reflective motivation for initiation were expressed in a theme ‘Necessity concerns balance’ underpinned by two sub-themes: ‘Lack of identification with being at risk’ and ’Safety concerns’. Having an optimistic outlook on pregnancy [47,48], women did not identify with the risk factors and therefore did not identify with someone who requires or takes medication [46,48,50].

*‘‘Women did not consider pre-eclampsia to be a serious disease*. *This led to a sense of optimism and reduced their sense of necessity in medication as there were no visible consequences of not taking the medication: “I kind of thought: If I’ll get that–I’ll get that*. *It wasn’t a big deal*.*”[47]*

*‘‘I think they put the stigma on people that are overweight*.*” [48]*

Lack of identification with a ‘medication taker’ exacerbated by a common negative perception of medicine use in pregnancy (unlike use of natural remedies), had a detrimental impact on initiation of aspirin [45,47–49]. As a result, women were not ‘buying into’ a concept of prevention. Safety concerns were exacerbated by difficulties to assess aspirin and inconsistent information about aspirin use in pregnancy (see physical and social opportunity section). Both themes were linked creating fine necessity-concern balance responsible for a decision-making process related to initiation of the treatment.

*‘‘Some participants said that they rather avoided taking ‘medication’ in general and especially during pregnancy*. *In this respect*, *aspirin ‘felt more like medication’ than calcium*, *as the latter was considered to be a ‘natural’ substance*.*” [49]*

*‘‘I didn’t like taking medications in general*.*” [47*,*50]*

*‘‘Some participants expressed concerns about lack of information on the risks of LDA: “I’m not sure I would [take aspirin] during pregnancy unless the data showed that it was safe*.*” [45]*

#### Reflective motivation: Facilitators

Explicit cognitive processes such as evaluation of past experience, and matching expectations [52] played facilitative role in the decision making related to initiation of aspirin. In this review cognitive processes involved in the decision making were aided by the following concepts: ‘Buying into prevention’, ‘Meeting expectations’, and ‘Taking control’.

#### Buying into prevention

Women arrived at a decision to take aspirin in pregnancy through reflective processes involving weighing benefits and disadvantages of aspirin use and accepting the idea of prevention.

*“I knew that I’d started aspirin very early and so I knew that hopefully that would have*, *you know*, *theoretically had an impact on the kind of placental development which I was hoping was gonna stand me in good stead …” [46]*

#### Meeting expectations

Having been prewarned about a need for aspirin prophylaxis, a follow up recommendation in antenatal period reinforced importance of this preventative strategy.

*“I knew that I’d started aspirin very early and so I knew that hopefully that would have*, *you know*, *theoretically had an impact on the kind of placental development which I was hoping was gonna stand me in good stead …” [46]*

*‘‘I knew exactly what was gonna happen at sort of what point cos I’d already been like pre-warned*.*” [46]*

#### Taking control

More women also felt that they should be in control of prevention taking ownership of their antenatal care.

*‘‘I think prevention is better than cure*.*” ‘‘…it was considered important that everyone*, *irrespective of one’s risk of pre-eclampsia*, *should get the option to make this choice: “Not giving people a choice is worse than the possibility of worrying them by telling*. *If you worry about it and there is something available*, *then at least you can do something about*.*” [49]*

#### Implementation of low dose aspirin

**Fig 3:**
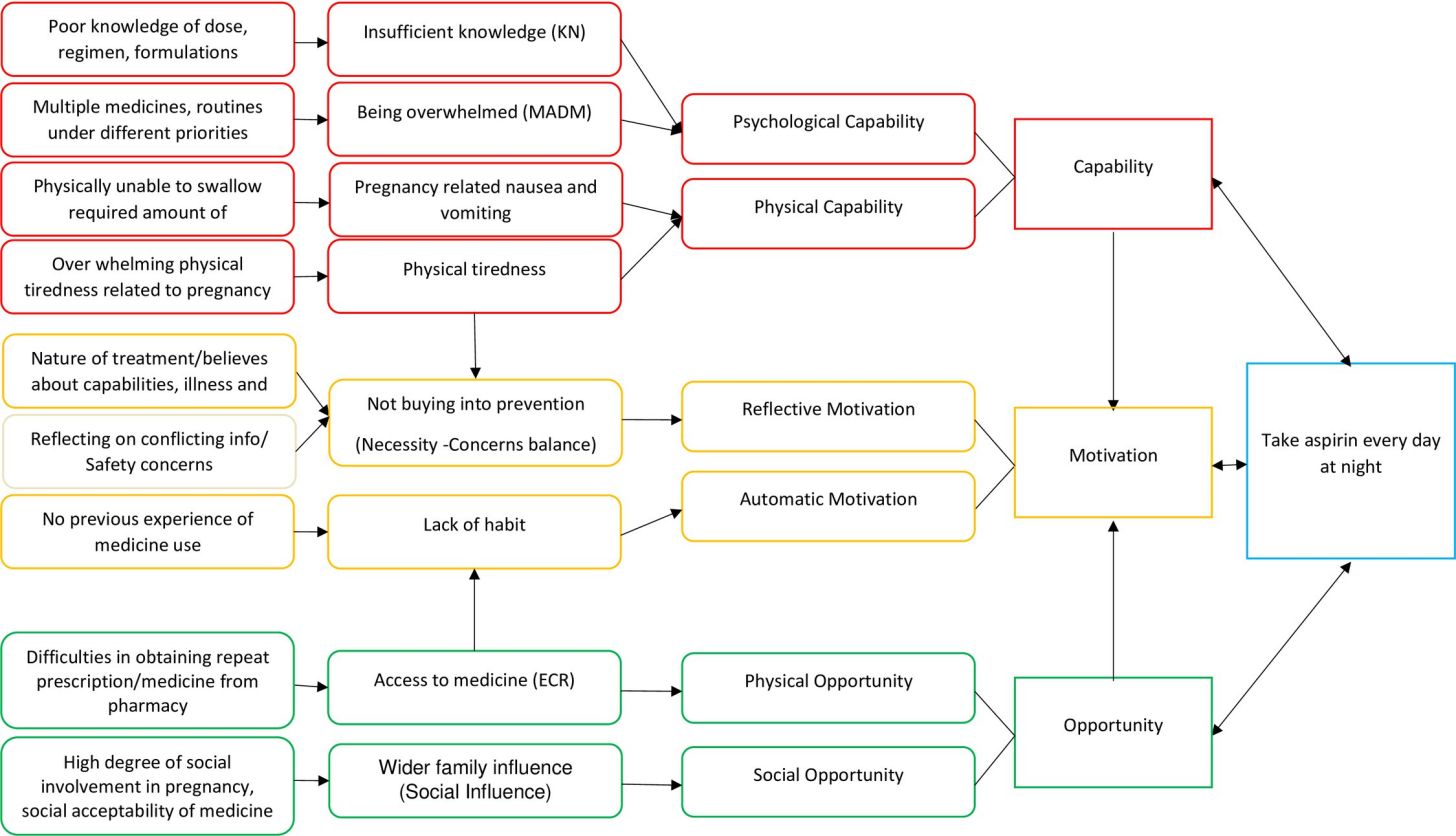
Barriers to implementation of aspirin.

The implementation phase of adherence is defined as adherence to the dosing regimen i.e., how closely actual dosing corresponds with the prescribed one. The main themes related to implementation process are illustrated in Fig 3.

#### Psychological capability: Barriers

Sense of ‘Being overwhelmed’ and ‘Insufficient knowledge’ acted as barriers to implementation under the psychological capability category.

#### Being overwhelmed

The overwhelming sensation present at the initiation, persisted throughout the implementation phase with women often being overwhelmed with the number of medicines they were required to take as well as by other routine tasks.

*“To be honest with you*, *I was taking a lot*. *There was a time where I was taking eight per day; that included things like Elevit*, *Vitamin D*, *my blood pressure medication and aspirin*, *yeah*, *so a lot*. *I had to take my diabetic medication*, *Aspirin*, *Macrolide*, *Folate and Vitamin D and Calcium so yeah it was hard to keep track of all of it*.*” [50]*

*“Erm*, *I have missed a couple of times*. *I tend to be fine when I’m at work cos I remember to take them when I’m at work but it’s on a weekend when I’ve got like my three-year-old and I’m trying to do the housework and keep everything going*, *it tends to be those days that I forget*.*” [46]*

#### Insufficient knowledge

Women were not aware of what dose or formulation of aspirin should be used.

*‘The women were not always advised on exactly how to take the medication; what formulation to take*, *when to take it or for how long*.*” [46]*

### Psychological capability: Facilitators

#### Behavioral regulation/strategies

To support regular use of aspirin women used various monitoring and planning strategies grouped under a facilitative theme ‘Behavioral regulation/strategies’.

*“I got a tablet box*, *like a daily one and like I put them in and I know if I haven’t taken them kind of thing*.*” (ASPQUA07); “I mean I’ve missed a couple of days*, *but it’s not been like every day*. *I’ve got an alarm set on me phone to remind us to take it …” [46]*

*“I put all my medications in my room in my drawer so I knew when I went to bed I pulled out the drawer and get all my medications out ready and yeah go to bed*. *I knew where all my medication was*, *and I had to take it*.*” [50]*

*“In addition to developing a routine*, *some women also used reminders*, *calendars and pill boxes to support adherence and establish new routines/habits*, *reducing unintentional non-adherence*.*” [48]*

#### Physical capability: Barriers

‘Pregnancy related nausea and vomiting’, and ‘Tiredness’ were themed under the physical capability category that hindered implementation.

#### Pregnancy related nausea and vomiting

This provides a context outlining physical circumstances in which pregnant women were advised to start aspirin. Women are advised to commence aspirin therapy at around 12 weeks gestation, at a time when pregnancy related sickness remains prevalent. Some women are not able to consume even small volumes of fluid, so consuming aspirin dissolved in water could prove a challenge to them. Yet, this is a very common formulation of aspirin that is dispensed to pregnant women.

*“I think there’s also been a couple of mornings*, *I think particularly early on when I was taking it*, *where I did feel quite sick in the morning and I was taking the dissolvable aspirin*, *and it did make me feel really queasy …” “I didn’t like taking it in water … it was like the after-taste of it …” [46]*

#### Tiredness

In addition, physical tiredness associated with pregnancy prevented women from sticking to their routines of taking aspirin.

*“When I got into bed and forgot to take all my medications*, *I went ‘I’m not getting back out of bed*. *I’m exhausted*. *Yeah, I’m in bed for the night now*. *I know later on I’m going to get out of bed a million times*. *No*, *I’m not getting out just to take medication*.*” [50]*

### Social opportunity: Barriers

#### Wider family influence

‘Wider family influence’ served as a key influence of the implementation. In a context of conflicting massages ‘Wider family influence’ served as a barrier to use of aspirin as advised. Negative influence caused by contradictory advice was amplified by family members and significant others who in turn have the ability to influence women’s decisions regarding the use of aspirin in pregnancy.

*“The chemist kept telling me that I should not take aspirin while I was pregnant despite my doctor’s advice*. *This made my husband and mother very concerned*, *and they discouraged me from taking the aspirin*.*” [50]*

### Social opportunity: Facilitators

#### Support from family members and HCPs

Support provided by family members and HCPs through reminding and reiterating the importance of keeping up with taking aspirin played an important facilitative role in supporting women to continue to take aspirin as advised.

*“I remember my doctor saying not to forget to take my medications*, *especially the aspirin*, *so actually I do recall her saying that to me and made it think it must be important for her to say that*.*” [50]*

*‘‘Many having strategies to help them remember their medication*, *which included phone reminders*, *pill boxes and often their partners*.*” [46]*

### Physical opportunity: Barriers

#### Access to medication

‘Access to medication’ was reported as a barrier to implementation as women had difficulties to replenish aspirin supply.

‘*‘Replenishing medication was really difficult*. *I took it to the doctors*, *but they didn’t put it on the prescription*, *so I then I had to ring up my hospital too and get my medication cos the doctors didn’t have it on their system*. *This happened a couple of times to be honest and I did go without medication for a few days because like it was so much of a hassle to try and get it …” [48]*

### Physical opportunity: Facilitators

#### Resources and flexibility within the health care system

Some studies reported that the availability of resources and flexibility within the health care system provided women with additional support at the implementation phase:

*‘‘My doctor spent a lot of time to talk to us about it and put our mind at ease*. *She also called the chemist after we left*.*” [50]*

### Automatic motivation: Barriers

#### Lack of habit

When implementing aspirin therapy in pregnancy one study reported that women had little or no habit of taking medicines making it harder to keep up with taking aspirin regularly:

*‘‘I think*, *perhaps*, *I am not in very much in the habit of taking things” [48]*

### Reflective motivation: Barriers

#### Necessity-concerns balance

‘Necessity-concerns balance’ remained a key theme withing reflective motivation category. It was supported by a number of sub-themes capturing nature of prevention treatment, beliefs about the disease and capabilities, and reflection on conflicting information was giving rise to further safety concerns. The concept of aspirin uses for PE prevention, rather than as a treatment of an existent condition, demonstrated to be a challenge for some women and prevented them from sticking to a regular treatment. Some women had little self-efficacy related to regular use of medicine, others were discouraged from taking aspirin upon reflection on conflicting information provided from different sources.

*‘‘I didn’t have any symptoms of high blood pressure I think it (taking aspirin) would have probably been more reassuring for somebody who has it because you can see a distinct difference between pre-aspirin*, *post-aspirin*.*” [47]*

*‘‘No symptoms would arise if I didn’t take it*, *kind of thing*. *Forgetting it didn’t lead to any incidents kind of … or symptoms I suppose*, *which would make you to have the medicine” [48]*

*‘‘I’m no good at taking tablets even the folic acid tablets and stuff I didn’t really take them*. *Yeah*, *I think I’m just generally bad at taking tablets [… ] I would give it a go but I would probably be the same …” [48]*

*“The negative impact of inconsistent messaging between HCPs was evident through the qualitative data in which women elaborated on how to information they obtained from multiple HCPs influenced their adherence with aspirin (both positively and negatively): The chemist told me that I should not take aspirin while I was pregnant despite my doctor’s advice*.*”[50]*

### Reflective motivation: Facilitators

#### Buying into a concept of prevention

On the other hand, good understanding of risks of the disease and potential benefits of aspirin summarised under ‘Buying into a concept of prevention’ theme facilitated adherence.

*“No I think because of what happened the first time*, *I was conscious*, *I knew I had to take it every day*, *to see if it helped”*. *[46]*

*“When I was told by the first doctor*, *I was still a bit skeptical and it’s only when I saw the second and third doctor*, *it sunk in and I thought*, *it must be important as they are all saying the same thing*. *It then made sense*. *It works well when doctors communicate the same thing*, *it gives us confidence*.*” [50]*

*“I remember my doctor saying not to forget to take my medications*, *especially the aspirin*, *so actually I do recall her saying that to me and made it think it must be important for her to say that*.*” [50]*

#### Early discontinuation of low dose aspirin

**Fig 4:**
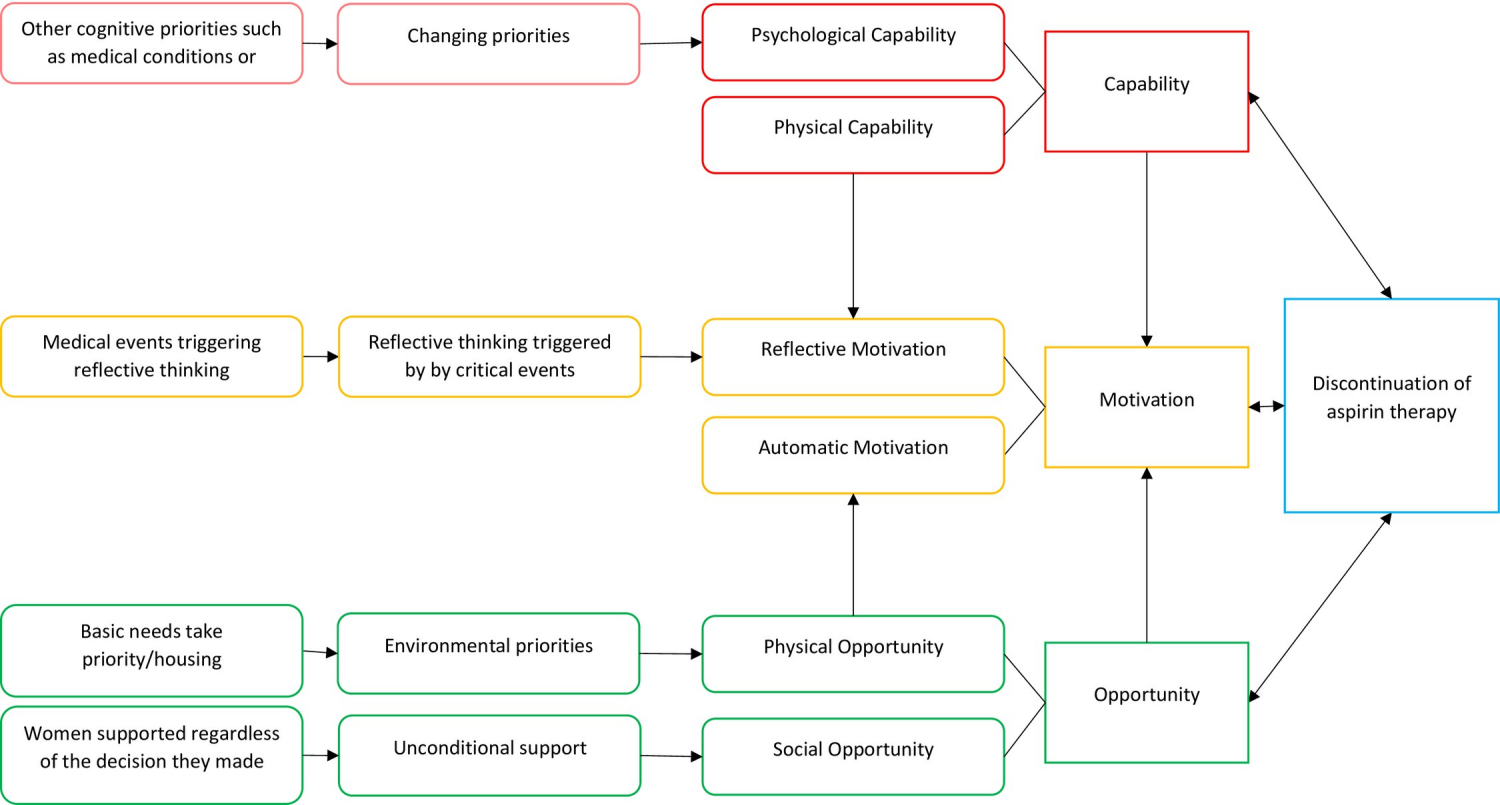
Barriers to timely discontinuation of aspirin in pregnancy.

Current clinical guidelines specify therapy discontinuation time: (ACOG, 2019; NICE, 2018). Therefore, non-adherence at discontinuation phase could be of two different types: early discontinuation that signifies complete discontinuation before the end date of prescribed therapy, and continuation of therapy beyond the end date. Although we searched for both types of non-adherence behaviour at the discontinuation phase, only evidence related to early discontinuation was found (Fig 4 shows the main themes related to the discontinuation process). Themes in this phase of adherence highlight the importance of a changing context in terms of ‘psychological’ and ‘environmental priorities’, changes to ‘Necessity-concerns balance’ with an addition to ‘Unconditional social support’ provided to pregnant women.

### Psychological capability influencing discontinuation

#### Changing priorities

Pregnancy is an ever-changing state in terms of physical and emotional status. The shift in priorities from a hypothetical risk of PE to developing a different health condition or having higher ranked emotional priority resulted in abrupt discontinuation when women were supposed to continue it intake.

*‘‘Events such as a change in medical condition*, *significant social issue such as housing problems and loss of a family member were coded as critical incidents affecting adherence*.*” [48]*

### Physical opportunity influencing discontinuation

#### Changing environmental priorities

Similarly, to psychological capability, environmental landscape often changes. Some women may experience critical events related to housing whether it is moving houses due to family expansion or loosing current housing. At this stage of life, basic environmental issues take priority over aspirin prophylactic therapy.

*‘‘I think the worst things that could happen*, *we also had to move out of our flat …” [48]*

### Social opportunity influencing discontinuation

#### Unconditional support

Interestingly, unconditional support available to women played a negative role in the process of discontinuation. Women were supported regardless of the decision they were making about the use of aspirin.

*‘‘and he (partner) said only you can make the choice if you’re not happy taking it then don’t take it*. *He says there’s plenty of women that go through pregnancies and don’t take it*.*” [48]*

#### Reflective motivation influencing discontinuation

Another reason for abrupt discontinuation was a disturbed balance between necessity and concerns. This happened as women were reflecting on critical medical events such as hospitalisation due to bleeding.

*‘‘But it was I think when I got there (to the hospital) and it was the bleed*, *it (aspirin) wasn’t the first thing that came to my mind*. *It was when they started asking me questions about what I take*, *what I was doing, you know … you kind of like go back and go; right*, *okay*, *I was asleep*, *so there’s nothing there … What do I take? They’re like: aspirin*, *why do you take aspirin? And I told them*. *And I was like*, *right*, *okay*, *and then it came to me*, *you know*, *it’s a blood thinner duh*, *duh*, *duh*, *duh*, *duh*, *duh and then you start*.*” [48]*

## Discussion

This systematic review and meta-synthesis of qualitative evidence aimed to compile the evidence on the barriers and facilitators of adherence to low-dose aspirin. Through extensive searches, we have identified six primary studies that investigate the issue of adherence using qualitative methods, all produced recently (since 2019). Considering the decades’ worth of clinical research into the use of aspirin in pregnancy and world-wide implementation of this preventative strategy, the issue of adherence seems to have been neglected. A recent global rise of awareness of issues with adherence to prophylactic aspirin use in pregnancy is reassuring and we hope will promote more effective use of this preventive treatment.

This systematic review has provided details of barriers and facilitators throughout the whole process of adherence to aspirin in pregnancy. By using phases of adherence [35] alongside the COM-B framework, this review identified gaps in knowledge regarding the discontinuation phase. No data being exposed and reported about prolonged use of aspirin beyond recommended timeframe. No data was found concerning to prolonged aspirin use beyond the recommended timeframe.

Many barriers identified in this review were not exclusive to adherence to prophylactic therapy in pregnancy but have additional dimensions related to pregnancy. The COM-B framework has been applied to medication adherence [53] using a wide range of qualitative and quantitative evidence related to influences of adherence in non-pregnant population [54–56]. Authors of ‘Applying COM-B to medication adherence: a suggested framework for research and interventions’ confirmed that the COM-B demonstrates a good match to existent evidence with a number of determinants extracted from the literature such as depression, substance abuse, marital status and forgetting mapping onto multiple COM-B components. Although in our review we had no influences related to depression, substance abuse, and marital status, forgetfulness was indeed linked to two COM-B components: related to cognitive overload (being overwhelmed) and lack of habit. Nevertheless, our review was able to identify key influences of adherence in pregnancy throughout the process of adherence that have critical importance to adherence to aspirin in pregnancy. ‘Insufficient knowledge’, ‘Necessity-Concerns balance’, ‘Access to medicine’, and ‘Social influence’ themes persisted as key influences across more than one phase of adherence.

Further, ‘Lack of habit’ during the implementation phase led to compromised adherence in women, even when their other key needs (‘Knowledge’, ‘Necessity-Concerns’, ‘Access to medicine’, ‘Social influence’) were being addressed.

Psychological capability was undermined by insufficient knowledge amongst women (as demonstrated in our review) but also amongst health care professionals [57–59]. This phenomenon is likely inherited by systemic exclusion of pregnant women from clinical trials in post Thalidomide scandal era [60]. Reflective processes described in the literature under the Necessity-Concerns framework [61], used by patients to weigh risks and benefits of medicine use, are more complex in pregnancy and rich beyond direct risks and benefits related to the mother herself. Risk-benefit consideration are further complicated by poor quality information and conflicting views expressed by health care professionals. It was highlighted in a recent study reporting pregnant women being denied or given negative comments about use of medicine that is needed to manage or prevent serious medical conditions in pregnancy [62]. Information related to medicine use in pregnancy shared via the internet also seemed to increase unjustified anxiety as fears of women are amplified by the global web [63], leading to an increase in concerns about the use of medicines in pregnancy in general.

Women’s concerns are further exacerbated by restricted access to aspirin. Despite being accessible in larger doses without a prescription over the counter, pharmacists necessitate a prescription to provide aspirin to pregnant women. This creates barriers to initiation and implementation of the treatment directly by making aspirin not accessible but also via initiation of reflective cognitive processes related to safety of the drug.

While concerns related to use of aspirin prevailed, there was evidence suggestive of reduced sense of necessity in preventative medicine as some women struggled to identify with the risk factors assigned to them by health care professionals. In a study of the psychological impact of providing women with risk information for PE, Harris et al described a typology of women differentiated by their reaction to allocation to a high-risk category in the absence of a reliable mitigation strategy: danger managers and fear managers. ‘‘Fear managers” unlike ‘‘danger managers” are embarking in an emotional path, feeling lack of control over disease prevention, and relying on medical professionals for reassurance with both ‘‘fear managers” and ‘‘danger managers” having low perception of risk [64]. Indeed, our review shows that despite being identified by HCPs at increased risk of PE, some women struggle to identify with risk allocation and some women feel lack of control over the condition [48]. This is likely due to their personal illness cognitions related to the nature of the disease, time-course, consequences, causal factors and control of the disease as described by the common-sense model of illness [65], that are based on misleading or little information available to women about PE, its risks, consequences, and preventative treatments.

‘Social influence’ by significant others and peers played an important role in adherence. Being pregnant involves a high degree of social circles involvement. Our review gathered evidence of involvement from partners, mother-in-law, grandmothers, friends, midwifes, neighbours and unknown individuals from internet forums, all being readily available to support women with their chosen course of action. This permissive culture during pregnancy calls for special attention of health care professionals and researchers with an interest in behavioural change.

Finally, adherence during the implementational phase is heavily relying on the development of new routines for medication intake. This review highlighted that women may be lacking on medication intake habits and need to be upskilled to develop new medication intake routines.

## Strengths and limitations

This is first systematic review and meta-synthesis of studies exploring issues of adherence to aspirin prophylaxis. Using the COM-B, we provide a comprehensive account of the barriers and facilitators related to adherence to aspirin in pregnancy. Close engagement of members of the public and representatives from relevant charities in this review allowed them to contribute to interpretation of the qualitative research, immerse in the scientific evidence available to date and enhanced their ability to engage in future work supporting intervention development and implementation.

This work has also benefited from input from an information scientist who supported the development of a comprehensive search strategy. In addition to comprehensive searches, we used dual screening processes to maximise identification of relevant literature [66]). Inclusion of grey literature in this review and meta-synthesis helped to widen literature searches and reduced the possibility of publication bias [67]. Although, this review attempted to conduct a comprehensive search of grey literature and utilised all available avenues for finding relevant work, a complex nature of searching for grey literature may mean that not all sources have been explored.

We acknowledge that this review is limited by the inclusion of only publicly available qualitative data, as full datasets (original transcripts) were not requested from the authors. This is due to potential issues arising from sharing qualitative data related to limited consent and data protection [68] that could have restricted data sharing. The ability to share data for some studies but not others could have created overrepresentation of one study data over those unable to share. Inclusion of second level constructs (authors’ interpretations), however, helped to expand and explain original data.

We recognise that inclusion of authors interpretations could have magnified researcher-related bias of the original research. Despite the fact that in some of the studies relationships between the researcher and participants were not adequately considered and the rigor of the data analysis not always easy to judge, we are confident in our synthesis findings as the presence of the themes in many cases were confirmed from different articles by different authors.

As well as having themes that had rich contribution from multiple papers, we did not exclude themes reported by a single source. The ‘Discontinuation’ phase is an example with a single author reporting on this phase. This is not reflective of a lesser importance of the discontinuation phase but likely related to the relatively small body of available evidence and to a very detailed approach taken in this meta-synthesis.

We would also like to highlight that two of the researchers in this review is also an author of two out of the six included studies. Although this added to expert knowledge of the field, it was important for the review team that the reviewer’s original studies were not over-represented. Researcher triangulation [69] was used to increase validity and to reduce the likelihood of over/under representation of the study’s data: a proportion of the included material was analyzed independently by a trained public contributor (EH) with stakeholders’ active participation and oversight of all stages of this systematic review and meta-synthesis. A step-by-step systematic review and framework synthesis were followed to increase the transparency of the review, with raw data available in (S5 and S6 Files).

Although qualitative research is not attempting to achieve generalizability, it provides an in-depth understanding of phenomena. This review demonstrated that similar barriers and facilitators of adherence to aspirin were described in different geographical as well as user settings. However, it is important to note that this review relies on data from high-income countries only and cannot be directly generalized to low-income countries’ contexts.

### Implications

Global breadth of this systematic review indicates that problematic adherence to aspirin is widely acknowledged but rarely addressed. Authors are aware of only two published interventions that aimed to improve adherence to aspirin through addressing educational components only [70,71]and one currently trailed intervention with a wider reach but of uncertain effectiveness [72]. As it is evident from this review that an addition of components that address access to medication, considers necessity-concerns balance effectively, utilises social support as well as helps to develop habits, could maximise effectiveness of future interventions. Currently, in absence of proven and effective interventions, health care providers could pay particular attention to the above-mentioned components while supporting women at increased risk of PE to engage in aspirin intake as a prophylactic measure.

## Future research directions

### Intervention development

Understanding the key influences on adherence to aspirin is the first essential step in intervention development [36]. Further work should involve a step-wise approach to intervention development: identifying intervention functions and determining content and implementation options. This process should be facilitated by key stakeholders to increase the acceptability of the future intervention and expedite its implementation in clinical practice [73].

### Further focus on timely discontinuation

In this review, no data related to the continuous administration of aspirin beyond the recommended time frame was found. This is not because the issue is nonexistent, but rather because it has not been adequately represented in qualitative research to date. Studies have primarily focused on aspirin prophylaxis in general, rather than addressing different phases of adherence. However, the issue of treatment continuation beyond the recommended time point has been highlighted in the Collaborative Low-dose Aspirin Study in Pregnancy (CLASP) trial, which was not included in this review as it does not contain qualitative data. In the CLASP trial, 53% of women extended aspirin treatment beyond the recommended timeframe [74]. Addressing this aspect of non-adherence becomes important as new evidence emerges related to the increased risk of bleeding in this cohort of women [75–77], as well as some evidence related to potential early discontinuation of aspirin [78]. As the international research community continues to advance knowledge related to the timing of discontinuation of aspirin treatment for PE prevention, it is crucial to highlight the need for exploring the reasons for the lack of discontinuation of this treatment and how these can be circumvented.

### Research in developing countries

Finally, all studies included in this review originate from high-income countries with well-established healthcare systems. Issues related to the implementation of aspirin use in low-income countries are not represented in the literature and are likely to differ significantly. The potential benefits of aspirin prophylaxis for reducing the risk of early onset of PE, which often leads to preterm delivery, are likely to be significantly higher in settings with limited availability of healthcare resources [79]. Exploring the utilisation of aspirin prophylaxis in low-income countries at the policy, healthcare provider, and service user levels, with the aim of implementing this treatment safely and effectively, could significantly impact the landscape of healthcare for women and babies in these regions.

## Conclusion

The COM-B framework allowed for detailed behavioural diagnosis of influences of adherence to aspirin in pregnancy based on the existing literature. This now provides a solid foundation for a process of intervention development with key target influences related to Psychological Capability (Inadequate knowledge), Physical opportunity (Access to medicine), Social Opportunity (Social Influences), Reflective and Automatic Motivation (Necessity-Concerns balance and Lack of Habit).

Having clear evidence of the influences of adherence, co-produced with key stakeholders, will improve and expedite the co-production of an evidence-based intervention that is feasible and acceptable for a wide range of stakeholders.

Although potential intervention functions could be suggested based on the results of this synthesis, additional co-production work is needed to define elements of a future intervention [80,81].

### Inclusive language acknowledgement for pregnancy-related terms

We recognize and affirm that when we use the term ’women,’ it is intended to be inclusive of all pregnant individuals. Our language is chosen with the understanding that gender identity is diverse, and we respect and acknowledge the experiences of all individuals who may become pregnant. By using ’women,’ we aim to honour the shared experiences of those who identify as women, as well as those whose gender identity may not align with the term but who share the experience of pregnancy.

## Supporting information

S1 File PRISMA 2002 checklist. (DOCX)

S2 File Search strategy. (DOC)

S3 File Inclusion and exclusion criteria. (DOCX)

S4 File Quality assessment CASP checklist. (DOCX)

S5 File Example of Linoit. (DOCX)

S6 File Data matrix. (XLSX)
