# Supplementary material for: Barriers and facilitators of adherence to low-dose aspirin during pregnancy: A co-produced systematic review and COM-B framework synthesis of qualitative evidence
Source: PLoS One. 2024 May 3;19(5):e0302720. doi: 10.1371/journal.pone.0302720 (PMC11068207; doi:10.1371/journal.pone.0302720)
Supplement: S2 File — (DOC) [file pone.0302720.s002.doc]

**S2: Search Strategy**

**Database: Ovid MEDLINE(R) 1946-**

**1**  Salicylates/
**2**  Aspirin/
**3**  aspirin.ti,ab,kf.
**4**  salicyl*.ti,ab,kf.
**5**  or/1-4
**6**  exp Pregnancy/
**7**  pregnan*.ti,ab,kf.
**8**  antenatal.ti,ab,kf.
**9**  ante-natal.ti,ab,kf.
**10**  or/6-9
**11**  determinan*.ti,ab,kf.
**12**  facilitat*.ti,ab,kf.
**13**  exp motivation/
**14**  motivat*.ti,ab,kf.
**15**  enabl*.ti,ab,kf.
**16**  empower*.ti,ab,kf.
**17**  inhibit*.ti,ab,kf.
**18**  incentive*.ti,ab,kf.
**19**  disincentive*.ti,ab,kf.
**20**  barrier*.ti,ab,kf.
**21**  (belief* or believ*).ti,ab,kf.
**22**  perceive*.ti,ab,kf.
**23**  perception*.ti,ab,kf.
**24**  attitude*.ti,ab,kf.
**25**  goal*.ti,ab,kf.
**26**  concordan*.ti,ab,kf.
**27**  nonconcordan*.ti,ab,kf.
**28**  adher*.ti,ab,kf.
**29**  nonadher*.ti,ab,kf.
**30**  (complian* or comply).ti,ab,kf.
**31**  noncomplian*.ti,ab,kf.
**32**  persistan*.ti,ab,kf.
**33**  (treat* adj5 refus*).ti,ab,kf.
**34**  (therap* adj5 refus*).ti,ab,kf.
**35**  (chang* adj5 (behavio?r or lifestyle)).ti,ab,kf.
**36**  ((modify or modifies or modifying or modification) adj5 (behaviour or behavior)).ti,ab,kf.
**37**  initiat*.ti,ab,kf.
**38**  implement*.ti,ab,kf.
**39**  discontinu*.ti,ab,kf.
**40**  exp "Patient Acceptance of Health Care"/
**41**  exp Patient Education as Topic/
**42**  patient education.ti,ab,kf.
**43**  exp Behavior/
**44**  (patient* adj5 agree*).ti,ab,kf.
**45**  concern*.ti,ab,kf.
**46**  harm*.ti,ab,kf.
**47**  effect*.ti,ab,kf.
**48**  outcome*.ti,ab,kf.
**49**  "necessity in medication".ti,ab,kf.
**50**  "necessity in treatment".ti,ab,kf.
**51**  or/11-50
**52**  exp qualitative research/
**53**  exp "surveys and questionnaires"/
**54**  interview/
**55**  grounded theory/
**56**  observation/
**57**  focus groups/
**58**  (qualitative or mixed metho* or interview* or grounded theory or ethnograph* or phenomenol* or observation* or focus group* or interpretive phenomenological analysis or IPA or action research or survey* or questionnaire*).ti,ab,kf.
**59**  or/52-58
**60**  5 and 10 and 51 and 59

**Database: Embase 1974-**
**Search Strategy:**
**1**  exp salicylic acid derivative/
**2**  aspirin.ti,ab,kf.
**3**  salicyl*.ti,ab,kf.
**4**  or/1-3
**5**  exp Pregnancy/
**6**  pregnan*.ti,ab,kf.
**7**  antenatal.ti,ab,kf.
**8**  ante-natal.ti,ab,kf.
**9**  or/5-8
**10**  determinan*.ti,ab,kf.
**11**  facilitat*.ti,ab,kf.
**12**  exp motivation/
**13**  motivat*.ti,ab,kf.
**14**  enabl*.ti,ab,kf.
**15**  empower*.ti,ab,kf.
**16**  inhibit*.ti,ab,kf.
**17**  incentive*.ti,ab,kf.
**18**  disincentive*.ti,ab,kf.
**19**  barrier*.ti,ab,kf.
**20**  (belief* or believ*).ti,ab,kf.
**21**  perceive*.ti,ab,kf.
**22**  perception*.ti,ab,kf.
**23**  attitude*.ti,ab,kf.
**24**  goal*.ti,ab,kf.
**25**  concordan*.ti,ab,kf.
**26**  nonconcordan*.ti,ab,kf.
**27**  adher*.ti,ab,kf.
**28**  nonadher*.ti,ab,kf.
**29**  medication compliance/
**30**  (complian* or comply).ti,ab,kf.
**31**  noncomplian*.ti,ab,kf.
**32**  persistan*.ti,ab,kf.
**33**  (treat* adj5 refus*).ti,ab,kf.
**34**  (therap* adj5 refus*).ti,ab,kf.
**35**  (chang* adj5 (behavio?r or lifestyle)).ti,ab,kf.
**36**  ((modify or modifies or modifying or modification) adj5 (behaviour or behavior)).ti,ab,kf.
**37**  initiat*.ti,ab,kf.
**38**  implement*.ti,ab,kf.
**39**  discontinu*.ti,ab,kf.
**40**  exp patient attitude/
**41**  exp Patient Education/
**42**  patient education.ti,ab,kf.
**43**  exp Behavior/
**44**  (patient* adj5 agree*).ti,ab,kf.
**45**  concern*.ti,ab,kf.
**46**  harm*.ti,ab,kf.
**47**  effect*.ti,ab,kf.
**48**  outcome*.ti,ab,kf.
**49**  "necessity in medication".ti,ab,kf.
**50**  "necessity in treatment".ti,ab,kf.
**51**  or/10-50
**52**  exp qualitative research/
**53**  exp health survey/
**54**  health care survey/
**55**  exp questionnaire/
**56**  exp patient health questionnaire/
**57**  exp interview/
**58**  grounded theory/
**59**  observation/
**60**  focus groups/
**61**  (qualitative or mixed metho* or interview* or grounded theory or ethnograph* or phenomenol* or observation* or focus group* or interpretive phenomenological analysis or IPA or action research or survey* or questionnaire*).ti,ab,kf.
**62**  or/52-61
**63**  4 and 9 and 51 and 62

**Database: APA PsycInfo**
**Search Strategy:**
**1**  exp Salicylates/ (561)
**2**  aspirin.ti,ab,id. (1176)
**3**  salicyl*.ti,ab,id. (271)
**4**  or/1-3 (1483)
**5**  exp Pregnancy/ (45223)
**6**  pregnan*.ti,ab,id. (52066)
**7**  antenatal.ti,ab,id. (4414)
**8**  ante-natal.ti,ab,id. (56)
**9**  or/5-8 (68683)
**10**  determinan*.ti,ab,id. (54596)
**11**  facilitat*.ti,ab,id. (182426)
**12**  exp motivation/ (163947)
**13**  motivat*.ti,ab,id. (178050)
**14**  enabl*.ti,ab,id. (96218)
**15**  empower*.ti,ab,id. (36509)
**16**  inhibit*.ti,ab,id. (149394)
**17**  incentive*.ti,ab,id. (20823)
**18**  disincentive*.ti,ab,id. (543)
**19**  barrier*.ti,ab,id. (93791)
**20**  (belief* or believ*).ti,ab,id. (215215)
**21**  perceive*.ti,ab,id. (279454)
**22**  perception*.ti,ab,id. (329727)
**23**  attitude*.ti,ab,id. (209462)
**24**  goal*.ti,ab,id. (214698)
**25**  concordan*.ti,ab,id. (9941)
**26**  nonconcordan*.ti,ab,id.
**27**  adher*.ti,ab,id.
**28**  nonadher*.ti,ab,id.
**29**  treatment compliance/
**30**  (complian* or comply).ti,ab,id.
**31**  noncomplian*.ti,ab,id.
**32**  persistan*.ti,ab,id.
**33**  (treat* adj5 refus*).ti,ab,id.
**34**  (therap* adj5 refus*).ti,ab,id.
**35**  (chang* adj5 (behavio?r or lifestyle)).ti,ab,id.
**36**  ((modify or modifies or modifying or modification) adj5 (behaviour or behavior)).ti,ab,id.
**37**  initiat*.ti,ab,id.
**38**  implement*.ti,ab,id.
**39**  discontinu*.ti,ab,id.
**40**  patient acceptance of health care.ti,ab,id.
**41**  patient education.ti,ab,id.
**42**  exp Behavior/
**43**  (patient* adj5 agree*).ti,ab,id.
**44**  concern*.ti,ab,id.
**45**  harm*.ti,ab,id.
**46**  effect*.ti,ab,id.
**47**  outcome*.ti,ab,id.
**48**  "necessity in medication".ti,ab,id.
**49**  "necessity in treatment".ti,ab,id.
**50**  or/10-49
**51**  exp qualitative methods/
**52**  exp surveys/
**53**  questionnaires/
**54**  exp interviews/
**55**  grounded theory/
**56**  observation methods/
**57**  (qualitative or mixed metho* or interview* or grounded theory or ethnograph* or phenomenol* or observation* or focus group* or interpretive phenomenological analysis or IPA or action research or survey* or questionnaire*).ti,ab,id.
**58**  or/51-57
**59**  4 and 9 and 50 and 58

**Web of Science**

1. **aspirin OR salicyl*** (Topic)
2. **pregnan* OR antenatal OR ante-natal** (Topic)
3. **(determinan OR facilitat* OR motivat* OR enabl* OR empower* OR inhibit* OR incentive* OR disincentive* OR barrier* OR belief* OR believ* OR perceive* OR perception* OR attitude* OR goal* OR concordan* OR nonconcordan* OR adher* OR nonadher* OR complian* OR comply OR noncomplian* OR persistan* OR initiat* OR implement* OR discontinu* OR "Patient Acceptance of Health Care" OR "Patient Education" OR behavior OR behaviour OR concern* OR harm* or effect* OR outcome* OR "necessity in medication" OR "necessity in treatment")**(Topic)
4. **treat* NEAR/5 refus*** or **therap* NEAR/5 refus*** or **chang* NEAR/5 (behavior OR behaviour OR lifestyle)** or **patient* NEAR/5 agree*** or **(modify OR modifies OR modifying OR modification) NEAR/5 (behaviour OR behavior)** (Topic)
5. **qualitative OR "mixed metho*" OR interview* OR "grounded theory" OR ethnograph* OR phenomenol* OR observation* OR "focus group*" OR "interpretive phenomenological analysis" OR ipa OR "action research" OR survey* OR questionnaire*** (Topic)
6. **3 or 4**
7. **1 and 2 and 5 and 6**

**Scopus**

( TITLE-ABS-KEY ( aspirin OR salicyl* ) ) AND ( TITLE-ABS-KEY ( pregnan* OR antenatal OR ante-natal ) ) AND ( TITLE-ABS-KEY ( qualitative OR "mixed metho*" OR interview* OR "grounded theory" OR ethnograph* OR phenomenol* OR observation* OR "focus group*" OR "interpretive phenomenological analysis" OR ipa OR "action research" OR survey* OR questionnaire* ) ) AND ( ( TITLE-ABS-KEY ( determinan OR facilitat* OR motivat* OR enabl* OR empower* OR inhibit* OR incentive* OR disincentive* OR barrier* OR belief* ) ) OR ( TITLE-ABS-KEY ( believ* OR perceive* OR perception* OR attitude* OR goal* OR concordan* ) ) OR ( TITLE-ABS-KEY ( nonconcordan* OR adher* OR nonadher* ) ) OR ( TITLE-ABS-KEY ( complian* OR comply ) ) OR ( TITLE-ABS-KEY ( noncomplian* OR persistan* ) ) OR ( TITLE-ABS-KEY ( initiat* OR implement* OR discontinu* OR "Patient Acceptance of Health Care" OR "Patient Education" OR behavior OR behaviour OR concern* OR harm* OR effect* OR outcome* OR "necessity in medication" OR "necessity in treatment" ) ) OR ( ( TITLE-ABS-KEY ( treat* W/5 refus* ) OR TITLE-ABS-KEY ( therap* W/5 refus* ) OR TITLE-ABS-KEY ( chang* W/5 ( behavior OR behaviour OR lifestyle ) ) OR TITLE-ABS-KEY ( patient* W/5 agree* ) OR TITLE-ABS-KEY ( ( modify OR modifies OR modifying OR modification ) W/5 ( behaviour OR behavior ) ) ) ) )

**CINAHL**

1. **TI,AB aspirin OR salicyl***
2. **TI,AB pregnan* OR antenatal OR ante-natal**
3. **TI,AB (determinan OR facilitat* OR motivat* OR enabl* OR empower* OR inhibit* OR incentive* OR disincentive* OR barrier* OR belief* OR believ* OR perceive* OR perception* OR attitude* OR goal* OR concordan* OR nonconcordan* OR adher* OR nonadher* OR complian* OR comply OR noncomplian* OR persistan* OR initiat* OR implement* OR discontinu* OR "Patient Acceptance of Health Care" OR "Patient Education" OR behavior OR behaviour OR concern* OR harm* or effect* OR outcome* OR "necessity in medication" OR "necessity in treatment")**
4. **TI,AB treat* N5 refus*** or **therap* N5 refus*** or **chang* N5 (behavior OR behaviour OR lifestyle)** or **patient* N5 agree*** or **(modify OR modifies OR modifying OR modification) N5 (behaviour OR behavior)**
5. **TI,AB qualitative OR "mixed metho*" OR interview* OR "grounded theory" OR ethnograph* OR phenomenol* OR observation* OR "focus group*" OR "interpretive phenomenological analysis" OR ipa OR "action research" OR survey* OR questionnaire***
6. **3 or 4**
7. **1 and 2 and 5 and 6**
