# Supplementary material for: Barriers and facilitators of adherence to low-dose aspirin during pregnancy: A co-produced systematic review and COM-B framework synthesis of qualitative evidence
Source: PLoS One. 2024 May 3;19(5):e0302720. doi: 10.1371/journal.pone.0302720 (PMC11068207; doi:10.1371/journal.pone.0302720)
Supplement: S4 File — (DOCX) [file pone.0302720.s004.docx]

**S4: Quality assessment using the CASP tool for assessment of qualitative studies.**

| **CASP Category** | | **Ahmed et al** | **Fenn et al** | **Shanmugalingam et al** | **Vestering et al** | **Vinogradov et al (1)** | **Vinogradov et al (2)** |
| --- | --- | --- | --- | --- | --- | --- | --- |
| 1 | **Was there a clear statement of the aims of the research?** | Yes | Yes | Yes | Yes | Yes | Yes |
| 2 | **Is a qualitative methodology appropriate?** | Yes | Yes | Yes | Yes | Yes | Yes |
| 3 | **Was the research design appropriate to address the aims of the research?** | Yes | Yes | Yes | Yes | Yes | Yes |
| 4 | **Was the recruitment strategy appropriate to the aims of the research?** | Yes | Yes | Yes | Yes | Yes | Yes |
| 5 | **Was the data collected in a way that addressed the research issue?** | Yes | Yes | Yes | Yes | Yes | Can't tell |
| 6 | **Has the relationship between researcher and participants been adequately considered?** | No | Yes | Can't tell | No | Yes | Yes |
| 7 | **Have ethical issues been taken into consideration?** | Yes | Yes | Yes | Yes | Yes | Yes |
| 8 | **Was the data analysis sufficiently rigorous?** | Can't tell | Yes | Can't tell | Yes | Yes | Yes |
| 9 | **Is there a clear statement of findings?** | Yes | Yes | Yes | Yes | Yes | Yes |
| 10 | **How valuable is the research?** | Yes | Yes | Yes | Yes | Yes | Yes |
