# Supplementary material for: Barriers and facilitators of adherence to low-dose aspirin during pregnancy: A co-produced systematic review and COM-B framework synthesis of qualitative evidence
Source: PLoS One. 2024 May 3;19(5):e0302720. doi: 10.1371/journal.pone.0302720 (PMC11068207; doi:10.1371/journal.pone.0302720)

**S5: Linoit example for data within initiation of the treatment for reflective motivation**


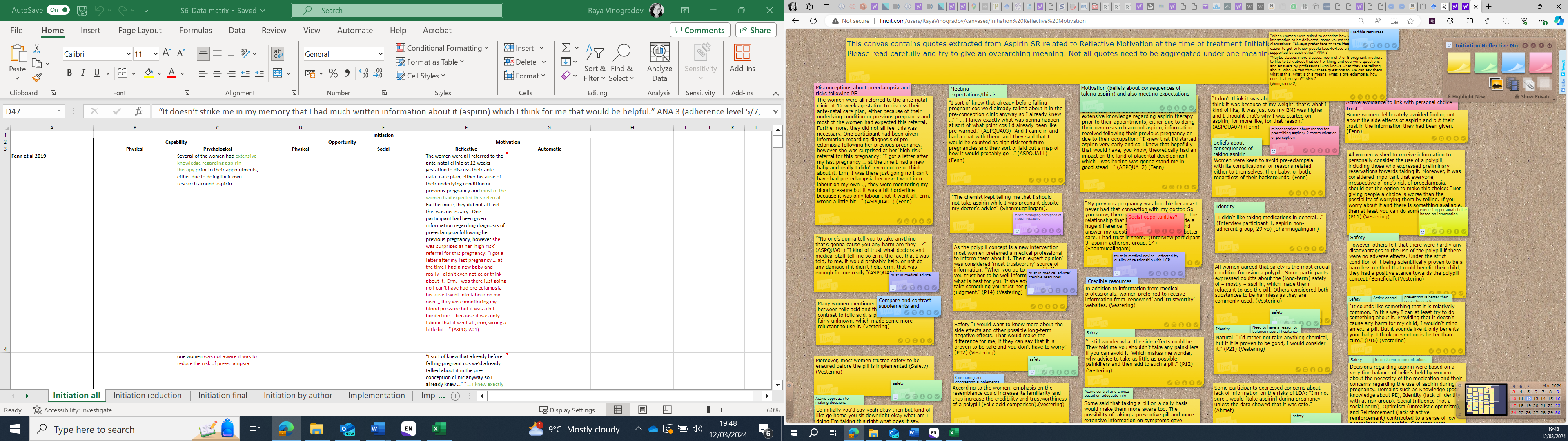


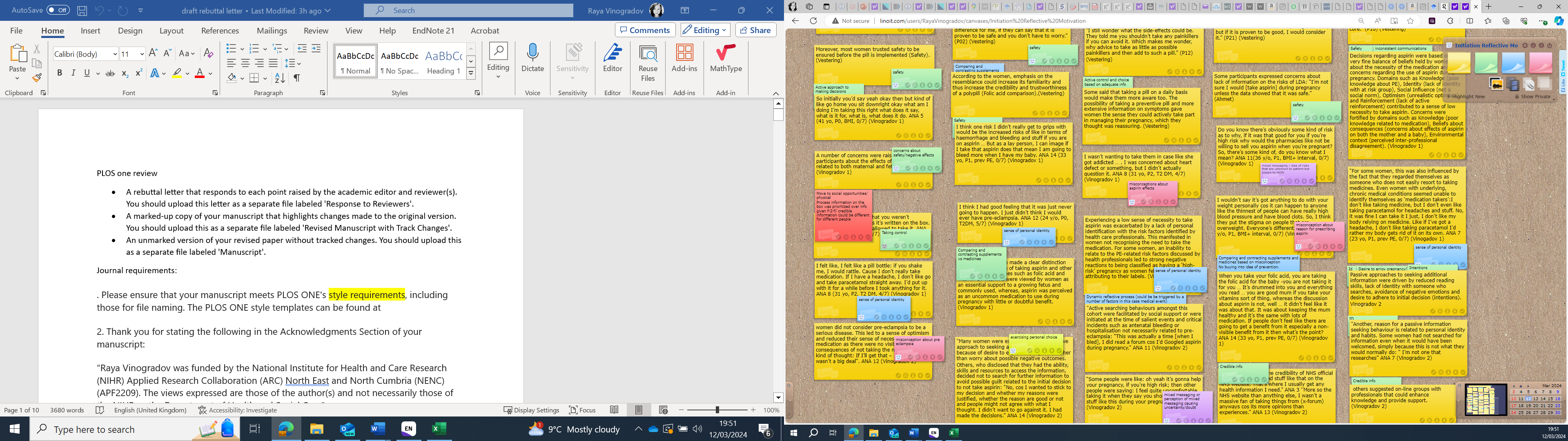


**Linoit example for data within initiation of the treatment for psycological capability**


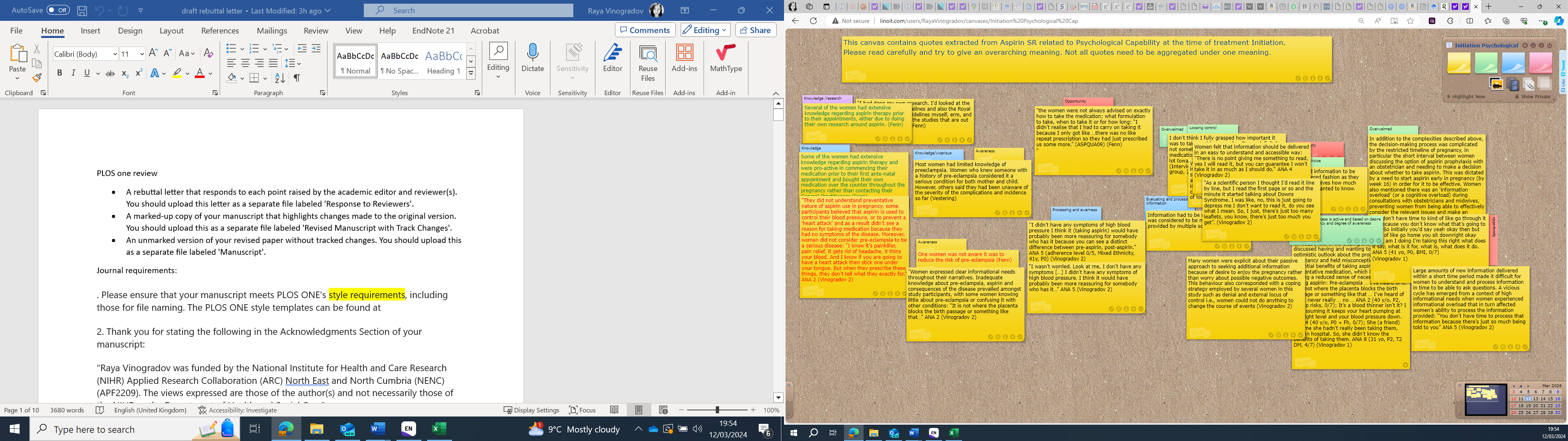

Supplement: S5 File — (DOCX) [file pone.0302720.s005.docx]
